# Evaluation of Estrogenic Activity of Licorice Species in Comparison with Hops Used in Botanicals for Menopausal Symptoms

**DOI:** 10.1371/journal.pone.0067947

**Published:** 2013-07-12

**Authors:** Atieh Hajirahimkhan, Charlotte Simmler, Yang Yuan, Jeffrey R. Anderson, Shao-Nong Chen, Dejan Nikolić, Birgit M. Dietz, Guido F. Pauli, Richard B. van Breemen, Judy L. Bolton

**Affiliations:** University of Illinois at Chicago/National Institutes of Health Center for Botanical Dietary Supplements, Department of Medicinal Chemistry and Pharmacognosy, College of Pharmacy, University of Illinois at Chicago, Chicago, Illinois, United States of America; Wayne State University School of Medicine, United States of America

## Abstract

The increased cancer risk associated with hormone therapies has encouraged many women to seek non-hormonal alternatives including botanical supplements such as hops (*Humulus lupulus*) and licorice (*Glycyrrhiza* spec.) to manage menopausal symptoms. Previous studies have shown estrogenic properties for hops, likely due to the presence of 8-prenylnarigenin, and chemopreventive effects mainly attributed to xanthohumol. Similarly, a combination of estrogenic and chemopreventive properties has been reported for various *Glycyrrhiza* species. The major goal of the current study was to evaluate the potential estrogenic effects of three licorice species (*Glycyrrhiza glabra*, *G. uralensis*, and *G. inflata)* in comparison with hops. Extracts of *Glycyrrhiza* species and spent hops induced estrogen responsive alkaline phosphatase activity in endometrial cancer cells, estrogen responsive element (ERE)-luciferase in MCF-7 cells, and *Tff1* mRNA in T47D cells. The estrogenic activity decreased in the order *H. lupulus* > *G. uralensis* > *G. inflata* > *G. glabra*. Liquiritigenin was found to be the principle phytoestrogen of the licorice extracts; however, it exhibited lower estrogenic effects compared to 8-prenylnaringenin in functional assays. Isoliquiritigenin, the precursor chalcone of liquiritigenin, demonstrated significant estrogenic activities while xanthohumol, a metabolic precursor of 8-prenylnaringenin, was not estrogenic. Liquiritigenin showed ERβ selectivity in competitive binding assay and isoliquiritigenin was equipotent for ER subtypes. The estrogenic activity of isoliquiritigenin could be the result of its cyclization to liquiritigenin under physiological conditions. 8-Prenylnaringenin had nanomolar estrogenic potency without ER selectivity while xanthohumol did not bind ERs. These data demonstrated that *Glycyrrhiza* species with different contents of liquiritigenin have various levels of estrogenic activities, suggesting the importance of precise labeling of botanical supplements. Although hops shows strong estrogenic properties via ERα, licorice might have different estrogenic activities due to its ERβ selectivity, partial estrogen agonist activity, and non-enzymatic conversion of isoliquiritigenin to liquiritigenin.

## Introduction

Because of an increased life expectancy in recent years, many women spend the last third of their lives in post menopause [1]. A drastic decline in circulating endogenous estrogen in menopausal women results in a number of symptoms including hot flashes, sleep disturbances, mood swings, vaginal dryness, and osteoporosis [2], [3], [4]. Hormone therapy (HT) has been the treatment of choice to alleviate menopausal symptoms. However, in light of the results published from the Women's Health Initiative (WHI), which demonstrated an increased risk of developing hormone dependent cancers, cardiovascular problems, and stroke among women taking HT, many women have turned to alternative therapies such as botanical dietary supplements to alleviate menopausal discomfort [5], [6], [7]. Currently, there is insufficient evidence on the efficacy of botanicals for menopausal symptom relief and their mechanisms of actions are not fully understood [8].

**Figure 1:**
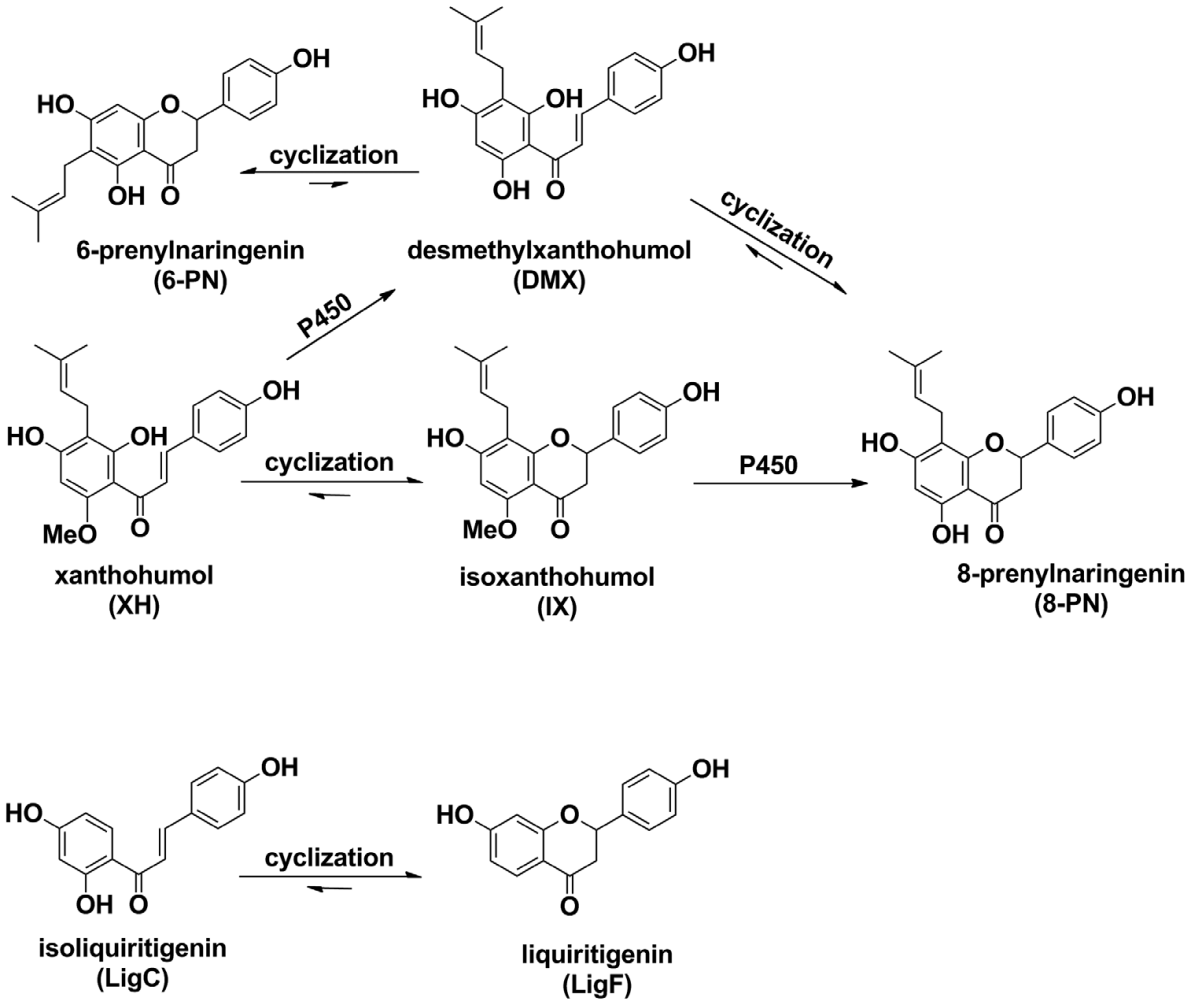
Estrogenic compounds in licorice and hops are formed from chalcones. Metabolism of bioactive compounds from **A**) hops and **B**) licorice.

Hops (*Humulus lupulus L.*) is a well studied botanical for women's health and a common constituent of dietary supplements, particularly in Europe [9], [10], [11], [12]. Hops and its phytoconstituents, including 8-prenylnaringenin (8PN) and its metabolic precursor chalcone, xanthohumol (XH) (Figure 1) have been studied for their estrogenic and chemopreventive properties [11], [12], [13], [14], [15], [16]. Hops have been shown to exert estrogenic activity in endometrial cancer (Ishikawa) and breast cancer (MCF-7) cells [12]. One of its bioactive compounds, 8-PN, the most potent phytoestrogen known to date [17], has been shown to be an equipotent ligand of estrogen receptor (ER) subtypes and exhibits estrogenic activity in hormone responsive cell-based assays as well as animal models [11], [12], [18]. However, XH, a metabolic precursor chalcone of 8-PN does not show estrogenic activity. It has been reported that, while a standardized extract of hops containing 8-PN did not increase uterine weight in ovariectomized Sprague-Dawley rats, 8-PN alone increased uterine weight and the height of luminal epithelial cells in animal models [11], [18]. While small amounts of 8-PN are present in most hops preparations, additional 8-PN can be biosynthesized *in vivo* through metabolism of XH (Figure 1). It has been reported that metabolic differences among individuals could impact the formation of 8-PN and likely the ultimate estrogenic responses generated by hops extracts [19]. On the other hand, XH, which does not have estrogenic properties, has been reported to possess chemopreventive potential, through the induction of detoxification enzymes [15].

Licorice root is one of the oldest and most frequently used botanicals in traditional Chinese medicine for improving health, curing injury or swelling, detoxification, and for women's health [20]. Today, licorice is mainly used as a flavoring and sweetening agent in tobacco industry, chewing gums, candies, toothpastes and beverages [20] and is one of the most popular components of menopausal dietary supplements in the United States [21], [22], [23]. Licorice has been studied for its estrogenic properties since 1950 [24], although the findings about its efficacy have not been conclusive [8]. There are more than 30 known licorice species in the world which differ genetically and biochemically. The different chemical profiles result in various biological activities and clinical potential among the species. The licorice species *Glycyrrhiza glabra* (*GG*), *Glycyrrhiza uralensis* (*GU*), and *Glycyrrhiza inflata* (*GI*) have been reported to contain various amounts of liquiritin, the glycosylated form of the dihydroflavanone, liquiritigenin (LigF) (Figure 1) and its precursor chalcone, isoliquiritigenin (LigC) (Figure 1), all of which have been reported to have estrogenic activity in vitro [25], [26], [27]. However, a comparative biological evaluation of distinct *Glycyrrhiza* species has not been conducted to date. In addition it is rarely clear which *Glycyrrhiza* species are present in menopausal dietary supplements and what species are better choices for these formulations in terms of estrogenic efficacy and safety. Studies that have reported estrogenic properties of licorice compounds, LigF and LigC, have not addressed the possible interconversion of these compounds which could strongly influence the interpretation of the estrogenic activities depending on the bioassay conditions.

In the present study, crude extracts of three licorice species, *GG*, *GU*, *GI*, as well as their active compounds, LigF and LigC, were examined for their *in vitro* estrogenic activity and were systematically compared with a spent hops (*Humulus lupulus*) extract in addition to its active constituents, 8-PN and XH. The conversion of LigC to LigF during bioassays was monitored by LC-MS and LC-UV. These results suggest that although licorice species are less estrogenic than hops, they contain an ERβ selective phytoestrogen, LigF and an estrogenic chalcone LigC which in turn can convert to LigF. The chalcone -> flavanone interconversion (LigC to LigF) in the case of licorice is non-enzymatic and therefore independent of metabolic variations among subjects. In contrast, with hops the two step conversion (XH to 8-PN) depends on CYP450 metabolism as well as gut microbiota [28], [29] which could differ among individuals with various metabolism characteristics. These data suggest that licorice extracts could benefit menopausal women due to moderate estrogenic activity, ERβ selectivity, and potentially a more predictable PK profile.

## Materials and Methods

### Chemicals and reagents

All chemicals and reagents were purchased from Fisher (Hanover Park, IL) or Sigma-Aldrich (St. Louis, MO), unless otherwise indicated. All media for cell culture and human recombinant ERα and ERβ were purchased from Invitrogen (Grand Island, NY). Fetal bovine serum (FBS) was purchased from Atlanta Biologicals (Norcross, GA). LigF and LigC were purchased from ChromaDex (Irvine, CA). 8-PN was synthesized [11] and XH was isolated from *H. lupulus* L.cv Nugget as described previously [30].

### Purity control of tested compounds

The purity and identity of all four tested chalcones/flavanones isomers, LigF/LigC and 8-PN/XH, were rigorously determined by orthogonal and complementary techniques using high resolution tandem mass spectrometric analysis (Waters Synapt QToF mass spectrometer) and quantitative^ 1^HNMR (qHNMR; spectra acquired at 298 K, using a 90° pulse experiment, on a Bruker Avance 600.13 MHz, equipped with a 5 mm TXI cryoprobe). The 100% qHNMR method [31] was applied to determine the purity of each compound and gave the following results: LigF (lot 12290-620) 96.9% w/w (calculated weight/ measured weight), LigC (lot 9265–726) 92.6% w/w, 8-PN 95.0% w/w, XH 96.5% w/w.

### Plant material, extraction, and characterization

Pelletized strobili of *Humulus lupulus* cv. Nugget were bulk extracted with food-grade ethanol. The fluid extract was dispersed in diatomaceous earth, dried, and bulk extracted with supercritical CO_2_ to yield two materials: the bitter acid extract (not used in this study) and the spent hop extract dispersed on the diatomaceous earth was used here. The spent hop extract was free of bitter acids. In preparation of the present experiments, the diatomaceous earth was removed by solubilization in methanol, filtration, and evaporation to dryness en vacuo. Quantitative LC-MS-MS analysis using authentic reference compounds as calibrants revealed that the spent hop extract contained 5.4% XH, 0.084% 8-PN, 0.076% 6-PN, and 0.65% IX (w/w % of the spent hops extract).

Samples of dried root materials of *Glycyrrhiza glabra* L. and *Glycyrrhiza. uralensis* Fisch. (Leguminosaea/ Fabaceae) were purchased from Indiana Botanical Gardens and from a local supplier at China Town (Chicago, IL), respectively. *Glycyrrhiza inflata* Batalin, a gift from Dr. Liang Zhao, Lanzhou Institute of Chemical Physics, was collected in Xinjiang province, China in 2008. A Botanical Center number was attributed to each sample which was identified through a series of macroscopic and microscopic analyses compared to authentic voucher samples deposited at the Chicago Field Museum.

Powdered roots from each of the three *Glycyrrhiza* species were exhaustively extracted by percolation with 100% methanol (MeOH, weight powder/volume of solvent: 1/20) at room temperature. Each extract was freeze-dried (mean extraction yield of 25% w/w (weight of extract/weight of root powder), and stored at −20 °C prior to any chemical or biological analysis. These crude extracts were compared and characterized through a combination of chromatographic techniques (High Performance Thin Layer Chromatography, High Performance Liquid Chromatography (HPLC) coupled with a photo-diode array (PDA) detector) and qHNMR analysis in order to obtain their characteristic chemical fingerprint. The marker compounds, LigF and LigC, as well as their glycosylated derivatives, liquiritin and isoliquiritin, were quantified in each *Glycyrrhiza* extract (10 mg/mL in MeOH HPLC grade) by UHPLC on an Acquity BEH C18 column (50×2.1 mm, 1.7 *μ*m) with PDA detection at 275 nm for the flavanones (LigF and liquiritin) and 360 nm for the chalcones (LigC and isoliquiritin). Samples (1 *μ*L injected) were eluted at 0.3 mL/min using the following gradient composition (A) H_2_O+0.1% formic acid and (B) acetonitrile +0.1% formic acid starting from 18% B during 2 min, to 30% B in 8 min and during 2 min, to 57% B at 17 min and during 1 min, and finally to 95% B at 22 min and during 3 min. Under these conditions, the retention time was 5.09 min for liquiritin, 10.56 min for liquiritigenin, 11.12 min for isoliquiritin, and 17.24 min for LigC. Linear regression equations were used to calculate the concentrations of LigC, LigF, Liquiritin and isoliquiritin (in mg/mL) in each extract. The calibration curves were corrected according to the purity of each standard as determined by qHNMR (100% method).

### Cell culture conditions

The Ishikawa cell line was provided by Dr. R. B. Hochberg (Yale University, New Haven, CT) and was maintained in Dulbecco's Modified Eagle Medium (DMEM/F12) containing 1% sodium pyruvate, 1% nonessential amino acids (NEAA), 1% glutamax-1, 0.05% insulin, and 10% heat-inactivated FBS [32], [33], [34]. The Ishikawa cell line is a well-established ERα (+) endometrial cancer cell line for the evaluation of estrogens and antiestrogens [32], [34]. Two days before treating the cells, the medium was replaced with phenol red-free DMEM/F12 medium containing charcoal/dextran-stripped FBS and supplements. Authentication of this cell line, via determination of the short tandem repeat (STR) profile [35] revealed its similarity with the Ishikawa cells according to the Health Protection Agency Culture Collection in the UK and also with the ECC-1 cells from the American Tissue Culture Collection, ATCC database (Manassas, VA). However, alkaline phosphatase was not inducible in ECC1 cells obtained from ATCC. Despite this controversy, we will keep the conventional name, Ishikawa, for this cell line throughout this paper. The MCF-7 cell line was purchased from ATCC. MCF-7 cells were grown in RPMI 1640 media containing 1% glutamax-1, 1% NEAA, 0.05% insulin, and 5% heat-inactivated FBS. Two days prior to treating the cells, the medium was replaced with phenol red-free RPMI 1640 medium containing charcoal/dextran-stripped FBS with acetone-washed activated charcoal (100mg/mL) at 4°C for 30 min and centrifuged at 4000 rpm for 15 min at 4°C. This step was repeated in triplicate. Extracts and compounds were not toxic to cells at the applied concentrations, under these experimental conditions. DMSO concentrations for all cell culture assays were below 0.1%.

### Detection of ER ligands using pulsed ultrafiltration LC-MS

A screening assay based on ultrafiltration mass spectrometry [36] was used to identify the ligands of ER present in licorice crude extracts. Briefly, 150 µg/ml of the methanol crude extract was incubated for 1 h at room temperature with 50 pmol of ERα or ERβ in binding buffer consisting of 50 mM Tris-HCl (pH 7.5), 10% glycerol, 50 mM KCl, and 1 mM ethylenediaminetetraacetic acid (EDTA) in a total volume of 50 µL. Identical control incubations in which denatured ER was substituted for active ER was used to correct for nonspecific binding of compounds to the ultrafiltration membrane and holder. After incubation, each mixture was filtered through a Microcon (Millipore, Bedford, MA) YM-30 centrifugal filter containing a regenerated cellulose ultrafiltration membrane with a 30000 MW cutoff and washed three times with 200 µL aliquots of ammonium acetate buffer (pH 7.5) at 4°C to remove the unbound compounds. The bound ligands were released by adding 400 µL of methanol/water (90∶10; v/v) followed by centrifugation at 10000×*g* for 10 min. The ultrafiltrates were dried under a stream of nitrogen, and the ligands were reconstituted in 50 µL of methanol/water (50∶50; v/v). Aliquots (10 µL) were analyzed using LC-MS, which consisted of a reverse phase separation on a Shimadzu (Kyoto, Japan) Shim-Pack XR-ODS III C_18_ (1.6 µm, 2.0 mm ×50 mm) column and mass spectrometric analysis on a Shimadzu LCMS- IT-TOF mass spectrometer. Both positive ion and negative ion mass spectra were acquired over the range *m/z* 100 to *m/z* 800. The ion source parameters for mass spectrometry included a capillary voltage of 3.5 kV, source block temperature 200°C, curved desolvation line temperature 200°C, and nebulizer gas flow of 1.5 L/min. The mobile phase consisted of a 5 min linear gradient from 5 to 100% acetonitrile in water with 0.1% formic acid.

### Estrogen receptor subtype (ERα/ ERβ) competitive binding assay

After identification of ER ligands in licorice crude extracts by mass spectrometry, competitive ERα and ERβ binding assays were used with [^3^H] estradiol based on the method of Obourn et al. [37] with minor modifications [13] to determine in vitro binding affinities of the ligands with the receptors. The reaction mixture consisted of 5 µL of extract in DMSO, 5 µL of purified human recombinant diluted ERα and ERβ (0.5 pmol) in ER binding buffer, 5 µL of “hot mix” [400 nM, prepared fresh using 95 Ci/mmol [^3^H] estradiol, diluted in 1∶1 ethanol:ER binding buffer; obtained from NEN life Science Products (Boston, MA)], and 85 µL of ER binding buffer. To correct for non-specific binding, a control containing all the added components except for the hot mix was considered. The incubation was carried out at room temperature for 2 h before 100 µL of 50% hydroxyapatite slurry (HAPS) was added. The tubes were incubated on ice for 15 min with vortexing every 5 min. The appropriate ER wash buffer was added (1 mL), and the tubes were vortexed before centrifuging at 10000×g for 5 min. The supernatant was discarded, and this wash step was repeated three times. The HAPS pellet containing the ligand-receptor complex was re-suspended in 200 µL of ethanol and transferred to scintillation vials. An additional 200 µL of ethanol was used to rinse the centrifuge tube. Cytoscint [4 mL/vial; ICN (Costa Mesa, CA)] was added, and the radioactivity was counted using a Beckman LS 5801 liquid scintillation counter (Schaumburg, IL). The percentage inhibition of [^3^H] estradiol binding to each ER subtype and the subsequent analysis were determined as were described previously [12].

### Induction of an estrogen-responsive alkaline phosphatase (AP) in Ishikawa cells

The protocol of Pisha et al. was used as described previously [34]. Ishikawa cells (5×10^4^ cells/well) were pre-incubated in 96 well plates in estrogen-free medium for 24 h. Test samples dissolved in DMSO, were added at different concentrations and the DMSO concentration was kept lower than 0.1%. To determine the anti-estrogenic activity, treatments were performed in the presence of 17β-estradiol (2 nM), well above its EC_50_. Plates were incubated at 37°C for 96 h. Cells were washed with PBS and lysed by adding 50 µL of 0.01% Triton X-100 in 0.1 M Tris buffer (pH 9.8) followed by a cycle of freeze and thaw at −80°C and 37°C, respectively. *p*-Nitrophenol phosphate (phosphatase substrate) (18 mM) was added to each well and the alkaline phosphatase activity was measured by reading the formation of *p*-nitrophenol at 405 nm every 15 s with a 10 s shake between readings for 16 readings using a Power Wave 200 microplate scanning spectrophotometer (Bio-Tek Instruments, Winooski, VT). The maximum slope of the kinetic curve for every experiment well was calculated. The fold induction of alkaline phosphatase for every treatment, compared to that of the estradiol control was represented as estrogenic activity and calculated as described previously [34]. Anti-estrogenic activity was stated as the fold induction of alkaline phosphatase compared to background induction control [34].

### Induction of estrogen responsive element (ERE) in MCF-7 cells

The Dual-Luciferase Reporter Assay System protocol from Promega (Madison, WI) was used to evaluate the activation of ERs through interaction with ERE at the promoter of estrogen responsive genes, resulting in the expression of the fused luciferase reporter. Briefly, MCF-7 cells grown in phenol-red free medium for 48 h, were trypsinized and re-suspended in serum-free medium at 1×10^7^ cells/mL followed by a 10 min incubation at room temperature with pERE (3 µg/mL) obtained from Dr. V. C. Jordan, Northwestern University [38], and pRL-TK (Promega) (1 µg/mL) in a 4 mm gap cuvette before electroporation at 950 µF and 250 V using the Gene Pulser Xcell (BioRad Laboratories, Hercules, CA). Transfected cells were diluted in serum containing medium and plated in 24-well plates (2×10^5^ cells/well). After 24 h, the cells were washed with PBS and treated with two concentrations of the crude extracts (2 µg/mL and 10 µg/mL) and two concentrations of the isolated compounds (0.1 µM and 1 µM), for an additional 24 h. 17-β-Estradiol (E2) (1 nM) was considered as the positive control. Cell lysates (20 µL) were placed in white Costar 96-well plates, before the injection of Luciferase Assay reagent (100 µL) followed by a 12 s read by a FLUOstar OPTIMA (BMG Lab Tech, Offenburg, Germany). To quench the firefly luciferase expression and activation of the renilla expression, Stop & Glo reagent (100 µL) was injected followed by a 12 s read. In order to exclude the errors in transfection efficiency, average read out of the luciferase activity was normalized to the average associated pRL-TK read outs (renilla activity). The results were converted into fold induction by normalizing to DMSO.

### Induction of estrogen-responsive gene mRNA in endometrial and breast cancer cells

Quantitative real-time polymerase chain reaction (qRT-PCR) was used to examine the modulation of the *Tff1* induction following treatment of T47D cells with the extracts and the related compounds. Experiments were performed four independent times in triplicates. T47D cells (4×10^4^ cells/mL) were preincubated in estrogen-free media for 72 h. Cells were treated with extracts (10 µg/mL), 8-PN (100 nM), LigF (5 µM), LigC (5 µM) in DMSO for 6 h. Total RNA was isolated using the TRIzol Plus RNA purification kit (Invitrogen) and quantitated by UV analysis at 260 nm. cDNA synthesis was performed using qScript cDNA synthesis kit (Quanta Biosceiences) in a total volume of 15 µL, containing 4 µL qScript reaction mixture (5X), 1 µL qScript RT, 10 µL nuclease-free water, and 5 µL of RNA sample. The reaction was carried out for 5 min at 22°C, followed by 42°C for 30 min and a 5 min incubation step at 85°C. The PCR and subsequent analyses were performed using the ABI StepOne Plus RT-PCR system (Applied Biosystems). Quantitation was performed using the TaqMan technology of Applied Biosystems. *Tff1* was evaluated using a predeveloped gene expression primer/probe set (Applied Biosystems' Assay on Demand). Briefly, the PCR reaction mixture was prepared in a total volume of 20 µL, containing 1 µL 20X TagMan Gene Expression Assay, 10 µL 2X TaqMan Gene Expression Master Mix, 4 µL cDNA template, and 5 µL RNase-free water. The reaction mixture was incubated at 50°C for 2 min followed by 10 min at 95°C. Polymerase chain reactions were performed in triplicate and consisted of 40 cycles with 15 s denaturing step at 95°C and 1 min annealing/extending step at 60°C each. The fluorescence signal was measured during the last 30 s of the annealing/extension phase. Following analysis, a fluorescence threshold value was set and threshold cycle (*C_t_*) values were determined. These values were used for further calculations. β-Actin was used as an endogenous control to correct for any differences in the amount of total RNA used for a reaction and to compensate for different levels of transcription during reverse transcription of RNA into cDNA. *Tff1* expression by treatments and controls were normalized to its respective β-actin expression levels. The final results were expressed as a fold induction, where the levels of *Tff1* observed in the DMSO-treated samples was defined as one.

### Isomerization of isoliquiritigenin to liquiritigenin

As shown in Figure 1, LigC forms a conversion equilibrium with LigF which influences the interpretation of the observations obtained by LigC. To evaluate this conversion and better define the compounds responsible for the observed estrogenic activity, LC-MS monitoring of the isomerization of these active principles in the cell-based assays was performed in parallel to every bioassay. One set of plates was considered for time 0, when there was no treatment and another set was considered for the final harvesting day for every experimental condition. The media exposed to cells were collected and extracted by adding sodium acetate buffer (100 mM, pH 5), followed by liquid-liquid extraction by water-saturated ethyl acetate. After evaporation of the solvent, the residue was dissolved in 50% methanol and analyzed using LC-MS or LC-UV to detect the conversion of LigC to LigF. When studying the stability of LigC in the competitive binding assay condition, the compound was incubated with the ER binding buffer before analysis.

### Statistics

The data are reported as the mean ± SD. Significant differences from control values were determined by one-way ANOVA with follow-up Dunnett test (P<0.05) using Graph-Pad Prism, version 5.00 for Windows, GraphPad Software.

## Results

### Quantification of chalcones/flavanones in *Glycyrrhiza* extracts

The marker compounds, LigF and LigC, as well as their respective glycosylated forms, liquiritin and isoliquiritin, were quantified in the crude MeOH extracts of *Glycyrrhiza* species (Table 1). The analyses showed that the *GU* extract contained the highest amount of LigF (0.16% w/w) compared to *GI* (0.06% w/w) and *GG* (0.05% w/w). Similarly, *GU* had the highest content of LigC (0.06% w/w) compared to *GI* (0.03% w/w) and *GG* (0.02% w/w). However, among the three licorice extracts, *GI* had the highest amount of glycosylated forms of LigF and LigC, liquiritin (5.47% w/w) and isoliquiritin (3.24% w/w), respectively. Nevertheless, the overall ratio of quantified flavanones versus the sum of flavanone and chalcones in *GU* (82% w/w) was considerably higher compared to *GI* (63% w/w) and *GG* (51% w/w). These data are consistent with the estrogenic activities of the various licorice extracts observed in the bioassays described below.

**Table 1:** Quantification of chalcones and flavanones in the *Glycyrrhiza* extracts.

	% in the crude extract (weight/weight dry extract)		
Quantified compounds	*GU*	*GI*	*GG*
Liquiritin (Glc-LigF)	3.23±0.13	5.47±0.18	1.64±0.08
Liquiritigenin (LigF)	0.16±0.05	0.06±0.05	0.05±0.03
Isoliquiritin (Glc-LigC)	0.69±0.15	3.24±0.78	1.64±0.16
Isoliquiritigenin (LigC)	0.06±0.03	0.03±0.01	0.02±0.01
Total aglycones: LigC +LigF	0.21±0.04	0.09±0.03	0.07±0.02
Total quantified flavanones (F)	3.39	5.53	1.69
Total quantified chalcones (C)	0.75	3.27	1.66
Total F/Total(F+C)	82%	63%	51%
Total C/Total(F+C)	18%	37%	49%

Values are expressed as mean ± SD of three independent analyses of each crude extract.

### Pulsed ultrafiltration mass spectrometric (PUF-MS) screening of the extracts

**Figure 2:**
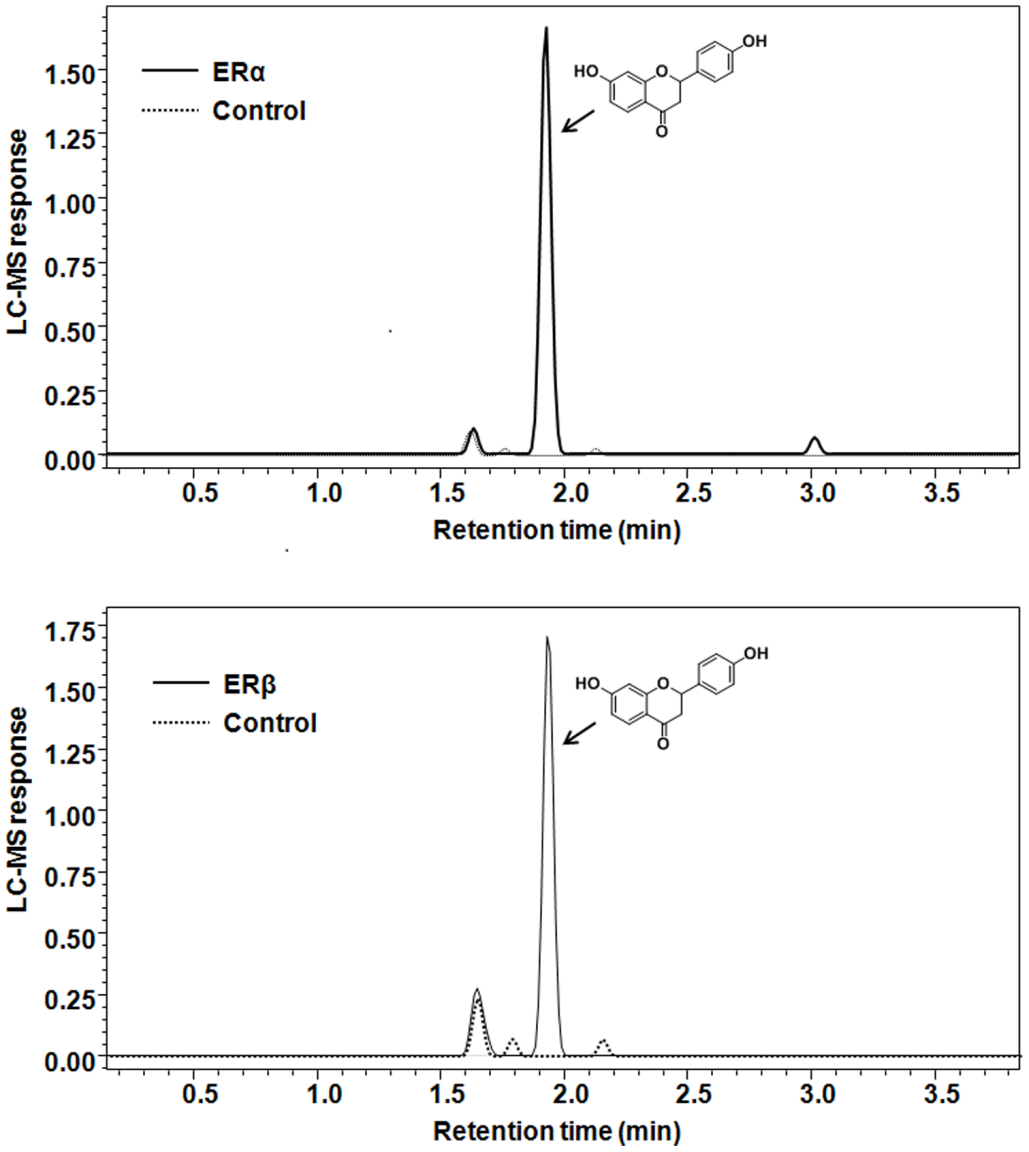
Liquiritigenin from licorice crude extract is the major ligand for ER subtypes in the pulsed ultrafiltration LC-MS analysis. Positive ion electrospray LC-MS chromatograms showing the ultrafiltration mass spectrometric screening of crude extract of *Glycyrrhiza uralensis*; *GU* to A) ERα and B) ERβ. Denatured ER was used as a control for non-specific binding and specific binding is indicated by increases in the chromatographic peak areas.

Pulsed ultrafiltration mass spectrometry is a rapid technique to identify active ligands for receptors in complex mixtures [39]. This method was used to find possible hits for ER subtypes in the crude MeOH extracts of the *Glycyrrhiza* species. Figure 2 shows that LigF was the only phytoconstituent which significantly enhanced the peak and bound to ERα and ERβ. Since PUF-MS analysis is a qualitative approach to define ligand-receptor interactions, a competitive binding analysis was also performed to quantitatively determine the affinity of the ligands for ERs.

### Relative affinity of the *Glycyrrhiza* compounds for ER subtypes

**Figure 3:**
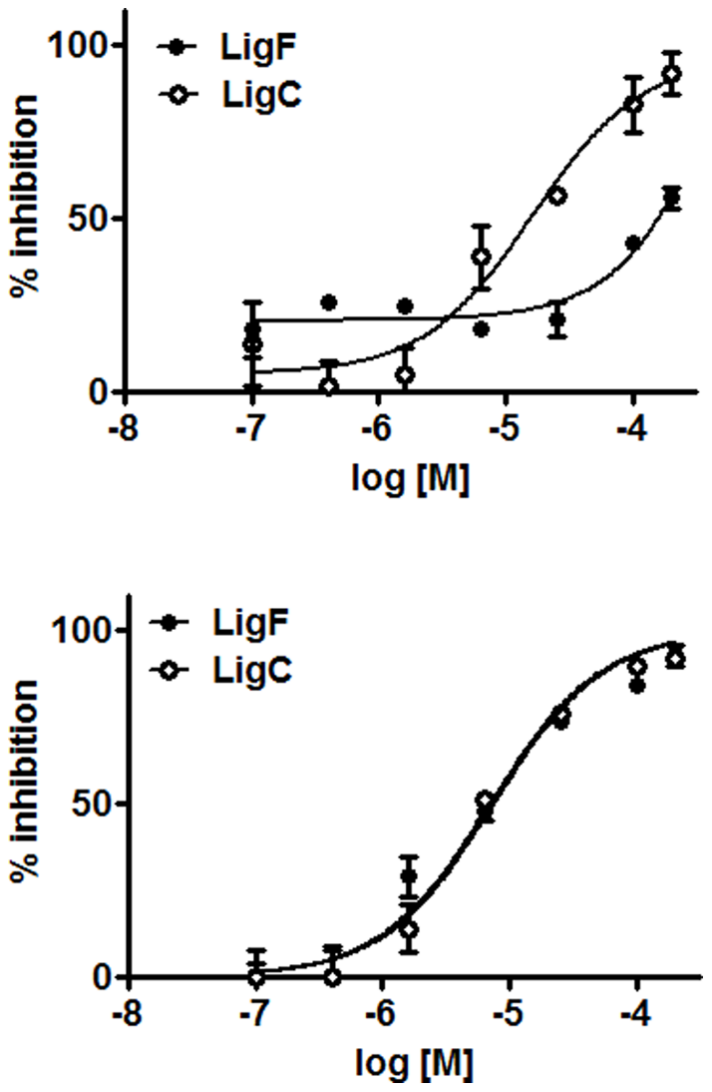
Liquiritigenin selectively binds to ERβ. Competitive ER binding using human recombinant A) ERα and B) ERβ.

Based on the screening results of PUF-MS analysis as well as literature reports [26], [40], competition of LigF and its precursor chalcone, LigC, with ^3^[H] estradiol for the ER subtypes was assessed to confirm and quantify the affinity of these compounds for ERs (Table 2, Figure 3). Both LigC and LigF had very similar affinities towards ERβ with IC_50_ values of 7.8 µM and 7.5 µM, respectively. However, LigF had a very weak affinity for ERα while LigC bound to ERα with an IC_50_ value of 16 µM. The selectivity of LigF for ERβ over ERα was 20-fold, which was comparable to the reports by Mersereau et al. [25] and Kupfer et al. (ERα IC_50_  = 2.8 µM, ERβ IC_50_  = 0.41 µM) [41]. Because different techniques were used the absolute IC_50_ values differed.

**Table 2:** AP induction, cytotoxicity, ER binding, and ERE-luciferase induction of licorice, hops and their isolated compounds.^a^

	17β-estradiol	*GU*	*GG*	*GI*	hops	LigF	LigC	8-PN	XH
ERE-luciferase fold induction^c,d^ (n = 9) MCF-7 cells	4.8±0.4	2.8±0.4	1.9±0.3	2.0±0.3	4.2±0.2	2.1±0.4	3.2±0.9	4.6±0.9	N/A
IC_50_^b^ (n = 9) ERβ	0.015±0.02	>50	>50	>25	27±3^i^	7.5±0.5	7.8±0.1	1.7±0.1^i^	N/A^i^
IC_50_^b^ (n = 9) ERα	0.021±0.03	> 200	>200	>200	15±3^i^	>200	16±1	0.51±0.07^i^	N/A^i^
Maximum AP fold induction Ishikawa cells	137±2.5	58.7±2.3	26.9±3.0	61±14.7	100±15	83.1±3.4	57.9±4.3	118±6.0	N/A
AP induction Ishikawa cells EC_50_^b^ (n = 9)	0.00019±0.00005	8.3±0.8	10.3±0.5	9.8±0.4	2.1±0.3	3.4±0.4	2.7±0.2	0.00665±0.00140	N/A

a Values are expressed as the mean ± SD of n determinations. Experimental details are described in the Materials and Methods section. ^b^ Values are expressed in µg/mL for extracts and µM for isolated compounds. ^c^ Fold inductions tested at 10 µg/mL for extracts (hops at 2 µg/mL) and 100 nM for the isolated compounds where DMSO was set to 1. ^d^ Ratio of the sum of the firefly and renilla luminescences. ^e^ N/A, not active. ^i^ J. Agric. Food Chem. 2005, 53, 6246–6253. ^j^J. Agric. Food Chem. 2001, 49, 2472–2479.

### Alkaline phosphatase induction in Ishikawa cells

**Figure 4:**
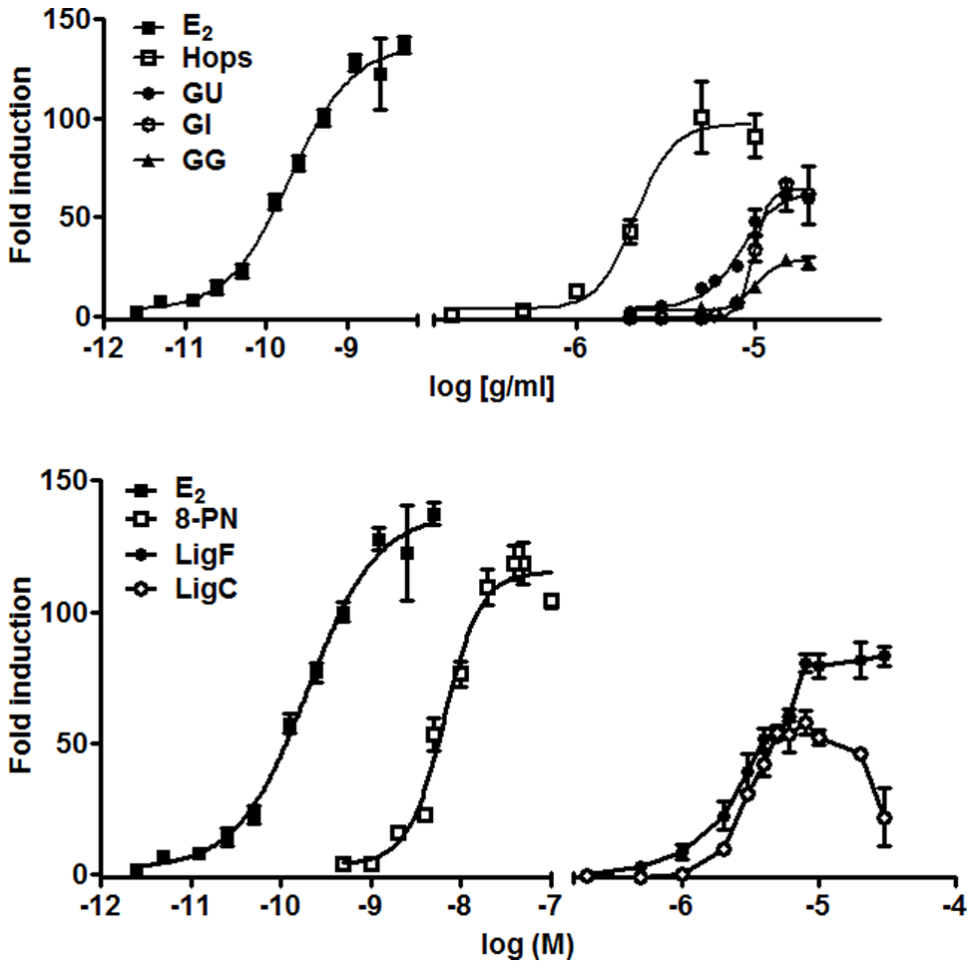
Different *Glycyrrhiza* species and their bioactive compounds are partial ER agonists with varied estrogenic potency and efficacy in Ishikawa cells. Induction of alkaline phosphatase in Ishikawa cells by A) crude extracts of *Glycyrrhiza glabra*; *GG*, *Glycyrrhiza uralensis*; *GU*, *Glycyrrhiza Inflata*; *GI* in comparison to hops and estradiol and B) isolated compounds liquiritigenin and isoliquiritigenin in comparison to 8-PN and estradiol. Results were normalized to DMSO and are shown as fold induction. Results are the means of three independent determinations. Dose-response curves were generated by non-linear regression analysis.

The Ishikawa cell line is a well-established ERα (+) endometrial cancer cell line for the evaluation of estrogens and antiestrogens [34]. Induction of alkaline phosphatase indicates estrogenic activity, while inhibition of alkaline phosphatase induction in the presence of 17-β-estradiol suggests a possible antiestrogenic effect. The crude MeOH licorice and spent hops extracts showed a dose-dependent induction of alkaline phosphatase (Table 2, Figure 4A). The EC_50_ values of the three *Glycyrrhiza* species were comparable: 7 µg/mL, 9.2 µg/mL, and 9.7 µg/mL for *GU*, *GI*, and *GG*, respectively. However, the maximum efficacy of *GU* and *GI* was around 60 fold, while that of *GG* was around 26 fold. On the other hand, the EC_50_ of hops at 2.1 µg/mL was consistent with previous reports [12] and was lower than that of the three *Glycyrrhiza* species, whereas its maximum efficacy was 100 fold. The relative EC_50_ ranking of the extracts were hops < *GU* < *GI* ≈ *GG,* while their relative maximum efficacy was hops > *GU* ≈ *GI* > *GG*. The EC_50_ values for the licorice purified compounds LigC and LigF were 2.7 µM and 3.4 µM, respectively (Table 2, Figure 4B) and their maximum efficacies were 58 fold and 83 fold, respectively. The EC_50_ of 8-PN from hops was 6.6 nM with 118-fold maximum efficacy, and XH was inactive, which was consistent with previous reports [12]. While the relative EC_50_ ranking of the isolated compounds was 8-PN << LigF ≈ LigC, their relative efficacies ranked: 8-PN > LigF > LigC. None of the extracts and isolated compounds showed antiestrogenic properties (data not shown). LigC showed a reduction in the estrogenic response at concentrations above 7.5 µM, which was associated with its cytotoxic effects at these concentrations. All samples were tested well below their LD_50_ concentrations for the Ishikawa cells (data not shown), unless otherwise stated.

### ERE-luciferase induction in MCF-7 cells

**Figure 5:**
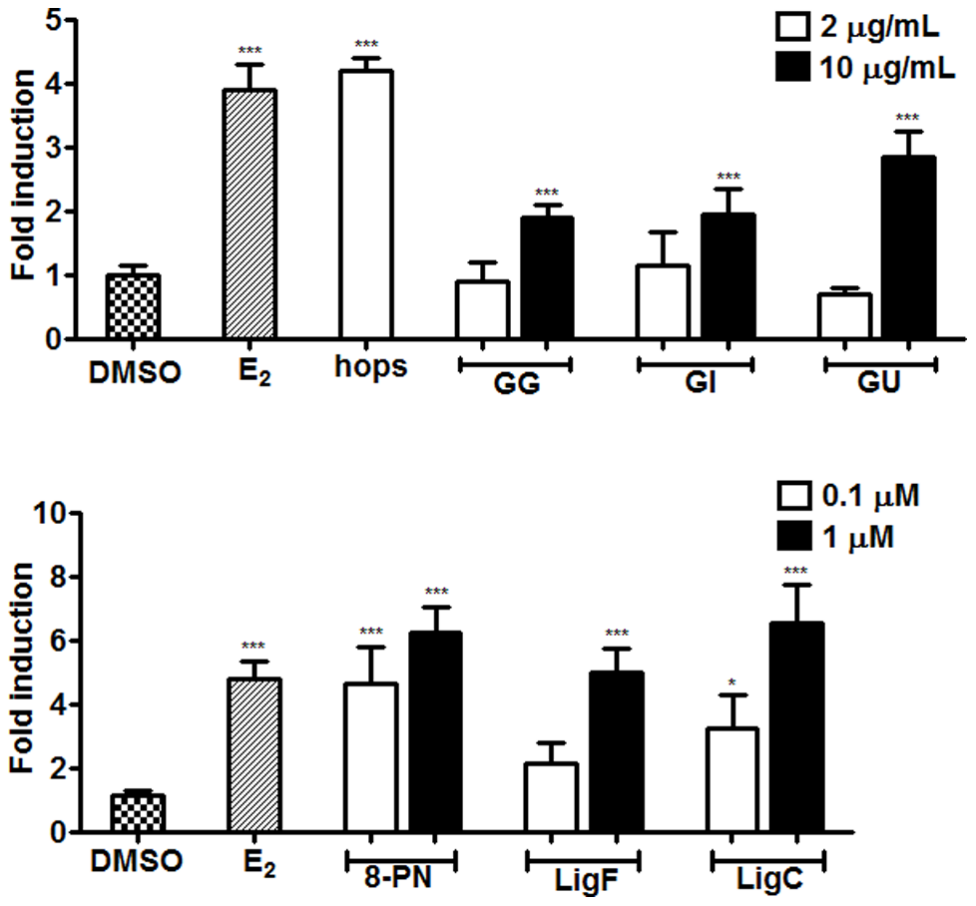
Different *Glycyrrhiza* species and their bioactive compounds induce ER dependent estrogenic response in MCF-7 cells. ERE-luciferase induction in ERα (+) MCF-7 cells by A) licorice and hops extracts and B) their respective compounds. Cells were cotransfected with pERE and pRL-TK 24 h before being treated with either extracts (2 µg/mL, open bars and 10 µg/mL, closed bars) or pure compounds (0.1 µM, open bars and 1 µM, closed bars). 17β-Estradiol (1 nM) was used as positive control. Since hops extract showed a considerable estrogenic activity at 2 μg/mL, higher concentrations were not tested. Chemiluminescence analysis was performed after 24 h. Results were normalized for transfection efficiency, and they are shown as a fold induction relative to the level observed in cells treated with vehicle only. Results are the means of three independent determinations in duplicates ± SD.

MCF-7 cells cotransfected with pERE-luciferase reporter and pRL-TK control were used to evaluate the ERE transcriptional activity of ERs in response to the applied treatments. The reporter response was evaluated relative to the control transfection and was presented as the fold induction after normalizing to the response of the DMSO treated cells (Table 2, Figure 5A). Induction of ERE-luciferase for hops (2 µg/mL) was 4-fold higher than that of DMSO. The *Glycyrrhiza* species were inactive at 2 µg/mL, but did demonstrate significant induction at 10 µg/mL. Both LigF and 8-PN showed induction of ERE-luciferase in MCF-7 cells (Figure 5B). On the other hand, while XH, the closely related chalcone of 8-PN, did not show any induction of ERE-luciferase in MCF-7 cells, LigC had a 7-fold induction at 1 µM, which was comparable to the induction levels by 8-PN (1 µM) and LigF (1 µM).

### Induction of estrogen responsive gene, *Tff1*, in T47D cells

**Figure 6:**
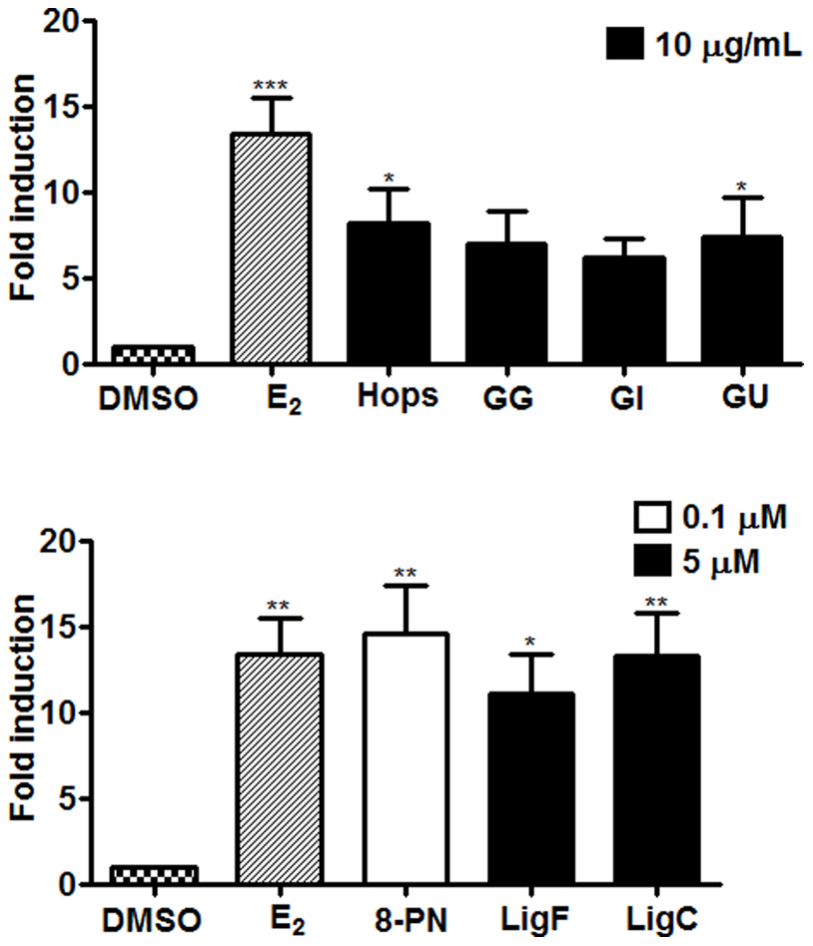
*Glycyrrhiza* species and their bioactive compounds induce the estrogenic marker,*Tff1* mRNA, in T47D cells. Estrogen responsive gene (*Tff1*) induction in T47D cells by A) licorice and hops extracts (10 µg/mL) and B) the related compounds 8-PN (100 nM), LigF (5 µM), and Lig C (5 µM). 17β-Estradiol (100 nM) was used as positive control. Results are the means of four independent determinations in duplicates ± SD.

Induction of trefoil factor 1(*Tff1)*, in ER (+) breast cancer T47D cells is a well-established tool to evaluate estrogenic activity of xenobiotics. Upon treating T47D cells with the extracts and the purified compounds, the total RNA was extracted and subjected to cDNA synthesis and qRT-PCR. The response was normalized to the corresponding effect of every treatment on β-actin gene induction and stated relative to the response of DMSO treated cells when DMSO response was considered as one. The results (Figure 6A) showed that *GU*, *GI*, and *GG* at 10 µg/mL induced *Tff1* in T47D cells, and induction of *Tff1* for the three *Glycyrrhiza* species and hops (10 µg/mL) were similar. Induction of *Tff1* by LigF (5 µM) was lower than that of 8-PN (0.1 µM), but the difference was not significant (Figure 6B). On the other hand, despite no *Tff1* activity by XH from hops, LigC from licorice induced *Tff1*, significantly.

### Analysis of the isoliquiritigenin-liquiritigenin isomerization *in vitro*

**Figure 7:**
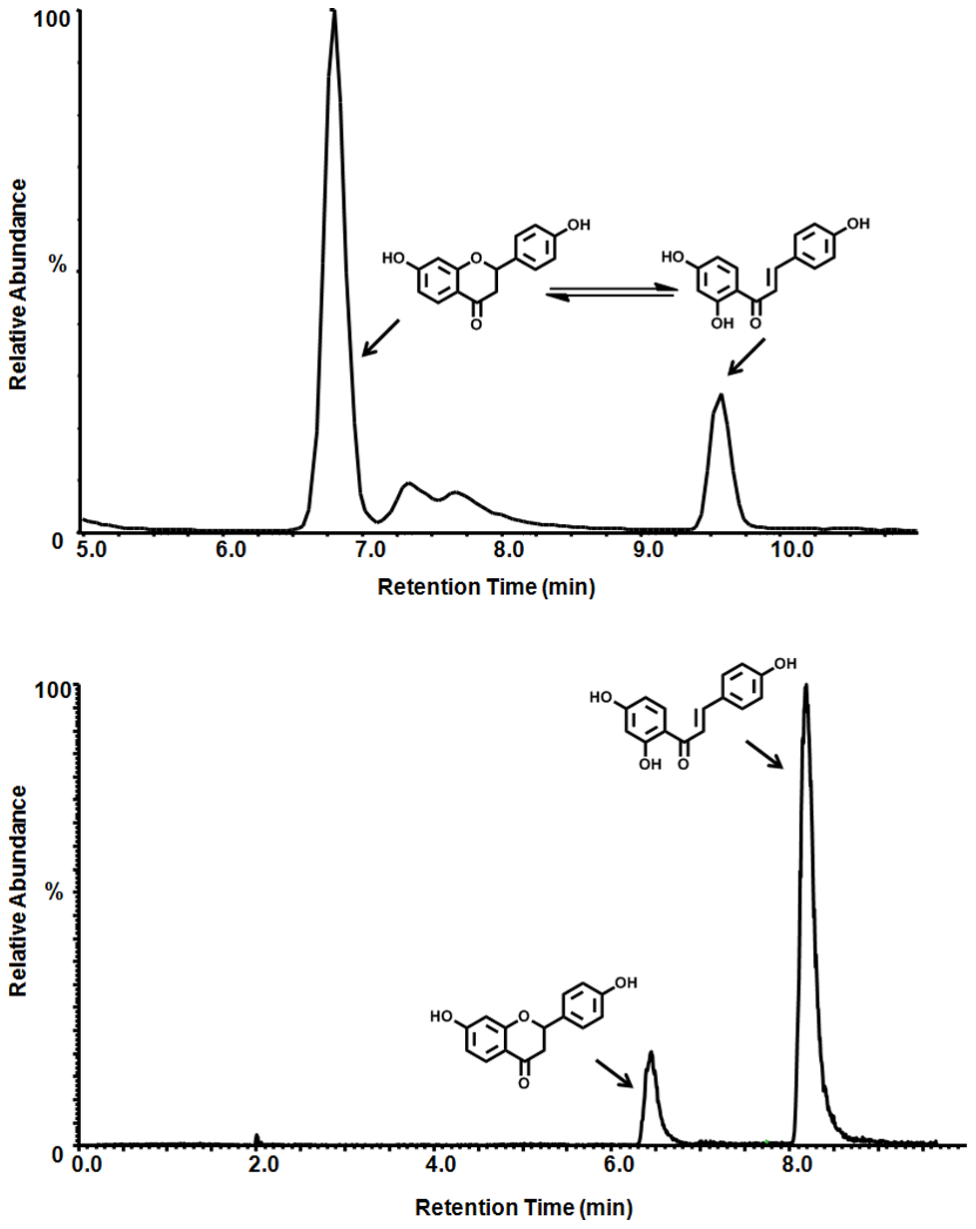
Chalcone-flavanone conversion in bioassays depends on time, pH, and temperature. LC-MS analysis of the isomerization of LigC to LigF: A) in the alkaline phosphatase induction assay conditions, 96 h incubation with cultured Ishikawa cells at 37°C. B) in the mRNA induction assay conditions, 6 h incubation with cultured T47D cells at 37°C.

Results from the alkaline phosphatase induction, ERE-luciferase induction, competitive binding to ERs, and estrogen responsive gene induction showed that LigC has estrogenic activity. The in situ isomerization of LigC to LigF (Figure 1) could potentially be involved in generating estrogenic responses observed with LigC. This hypothesis was confirmed by LC-MS analysis of the cell media of the alkaline phosphatase induction assay after 96 h which showed a significant reduction in the LigC content and a corresponding increase in LigF formation (Figure 7A). However, LC-MS analysis of the cell media of the mRNA induction assay after 6 h did not show a significant formation of LigF from LigC (Figure 7B) which indicates the role of incubation time in this 37°C conversion reaction. LC-UV analysis of the ER binding buffer in the competitive binding assay after 2 h incubation at room temperature did not show any isomerization of LigC to LigF (data not shown), which emphasizes the effect of incubation time in addition to temperature on the stability of LigC [42]. These data suggest that LigC could activate estrogenic responses on its own, although formation of the isomerization product, LigF, likely also contributes to observed estrogenic properties of LigC.

## Discussion

Previous studies on licorice extracts have primarily focused on the characterization of estrogenic properties of *GG,* because it is the most widely used licorice source material and most likely to be found in botanical supplements for women's health in North America [13], [43], [44], [45]. Previous findings [13] and the present work have demonstrated that *GG* does not have a strong estrogenic activity and, in fact, *GU* and *GI* are more estrogenic (Table 2).

Nevertheless, similar estrogenic potencies were observed for the three *Glycyrrhiza* species in the alkaline phosphatase induction assay in ERα (+) endometrial cancer cells (Table 2, EC_50_'s, Figure 4A). The estrogenic activity of the *Glycyrrhiza* species was confirmed in the ERE-luciferase assay in MCF-7 cells (Figure 5), as well as in the gene induction assay in T47D cells (Figure 6) suggesting that licorice has estrogenic properties in different estrogen sensitive tissues. These data suggest that the estrogenic active principle is likely the same among the species. This observation was consistent with the findings of the pulsed ultrafiltration LC-MS study that showed LigF as the ER ligand in all *Glycyrrhiza* crude extracts (Figure 2).

The pronounced difference in the efficacy of the estrogenic responses (Table 2, maximum AP) might be attributed to the varied amounts of LigF and its precursor chalcone (LigC) in the three investigated species (Table 1). Previously, Kondo et al. [27] had shown that *GU* has the highest content of LigF (0.11%) and its glycosylated form, liquiritin (1.68%), among the three investigated licorice species. Similarly, quantitation of the chalcone/flavanone ratio in each *Glycyrrhiza* extract (Table 1) revealed that *GU* contained the highest amount of LigF (0.16% w/w) compared to the other extracts (0.05% w/w for GG and 0.06% w/w for *GI*). Total LigF and LigC content of *GU* (0.21% w/w) was more than twice as high as that of *GI* (0.09% w/w) and *GG* (0.07% w/w). Moreover, the total flavanone content of *GU* extract (82% w/w) was significantly higher than that of *GI* (63% w/w) and *GG* (51% w/w) extracts, all explaining the higher estrogenic activity of *GU* extract.

*GG* has a higher ratio of chalcone content over total flavanones and chalcones (49% w/w) in comparison to *GI* (37% w/w) and *GU* (18% w/w). Chalcones with an accessible Michael acceptor/electrophilic moiety, such as LigC and XH, can interact with cysteine residues of cellular proteins and activate protective responses such as apoptosis [46]. XH can switch on some cytoprotective mechanisms including activation of detoxification enzymes and can lead to cytotoxicity at higher concentrations [15]. Recent studies have demonstrated that LigC induces apoptosis in different cell lines [47], [48]. Therefore, LigC might be responsible for the observed cytotoxicity of *GG* at concentrations >10 µg/mL (data not shown). LigC as a Michael acceptor can activate cellular chemopreventive processes [26], [49], [50], [51], [52]. However, in one animal study it has been reported that LigC enhanced breast tumor growth [53], which might be associated with its estrogenic properties or conversion to the estrogenic compound LigF.

Isomerization of chalcones to flavanones is a well established chemical phenomenon (Figure 1) [54], [55], [56], [57]. However, this conversion has often been neglected when studying the biological properties of chalcone/flavanone pairs such as LigC/LigF in bioassays. LC-MS analysis of this isomerization under bioassay conditions (Figure 7) in addition to a report by Simmler et al. [42] led to the conclusion that the cyclization is dependent on the pH, temperature, and incubation time. After 96 h incubation at 37°C with Ishikawa cells, LigC was mostly converted to LigF (Figure 7A); however, after 6 h incubation with T47D cells, the conversion was limited (Figure 7B). In case of the competitive ER binding assays, the analysis after 2h incubation at room temperature did not show a conversion of LigC to LigF, suggesting the ability of LigC to bind to ER subtypes (Figure 3) without converting to LigF (data not shown). Therefore, the active estrogenic compound(s) in licorice preparations could be both LigF and LigC, depending on the bioassay conditions. The conversion of LigC to LigF could be more pronounced under physiological conditions and needs to be evaluated *in vivo*.

LigF and LigC from licorice were compared with 8-PN and XH from hops in a variety of estrogenic assays. While, LigF selectively binds to ERβ confirming previous reports [25], [41], 8-PN has high affinity for both ER subtypes [12]. LigF has also been reported to selectively activate ERβ in functional assays [25], [58] which shows not only its selective binding towards ERβ, but also an activating role for ERβ dependent pathways. Selective activation of ERβ by estrogenic ligands has been reported to be correlated with down-regulation of ERα activities, including proliferation [59]. Therefore, selective activation of ERβ by LigF might present a safer mode of estrogenic activity of licorice, while hops and its constituents do not possess this preferential ERβ modulatory effect.

In the present study, LigF showed estrogenic activity in alkaline phosphatase induction assay in Ishikawa cells at micro molar concentrations and in a partial agonistic manner, while 8-PN was active in nano molar ranges with an efficacy close to the full agonist, E_2_. However, the activities of LigF in ERE-luciferase induction in MCF-7 cells and *Tff1* mRNA induction in T47D cells were similar to the activities observed with 8-PN, which might be related to cell type/signaling pathway specific activities of these compounds. LigC, the precursor chalcone of LigF was an equipotent ligand of ER subtypes in competitive binding assays, exhibited partial agonistic estrogenic activity in alkaline phosphatase induction assay in Ishikawa cells, and induced ERE-luciferase in MCF-7 cells as well as *Tff1* mRNA in T47D cells. However, XH, the precursor chalcone of 8-PN from hops, did not bind to ERs [12] or exhibit any estrogenic effects in corresponding bioassays. These data are consistent with previous studies which demonstrated that LigC is able to bind to the ER binding pocket and activate estrogenic responses [40], [52]. XH does not have this property and requires metabolism to convert to the estrogenic entity, 8-PN (Figure 1).

Hops extract exhibited higher estrogenic potency and efficacy in the alkaline phosphatase induction assay and higher activity in the ERE-luciferase induction assay compared to the licorice species. The stronger estrogenic activities of hops could be associated with the presence of 8-PN and its precursors in hops. Moreover, the licorice extract showed partial agonistic behavior, which could be a therapeutic advantage as partial agonists are better tunable agents for their respective receptor signaling pathway [60]. Partial agonists can play the role of antagonists when a full agonist is present and can also work more selectively. These properties make licorice and its active constituents an attractive target for further characterizations of their estrogenic activity.

In conclusion, these data show that *Glycyrrhiza* species have different estrogenic activities with *GU* showing the highest estrogenic properties. This further emphasizes the importance of precise labeling and definition of plant species in botanical supplements. Licorice and its compounds have partial agonistic estrogenic activities, LigF is an ERβ selective ligand and LigC shows dual estrogenic/chemopreventive activities. LigC and LigF are easily interconvertible without enzymatic metabolism [42]. All these properties suggest that licorice might have more moderate, potentially safer, and more predictable estrogenic activities than hops. Therefore, standardized licorice preparations could be considered as an option for menopausal women. Nevertheless, before estrogenic herbal supplements such as licorice are recommended, *in vivo* safety studies are necessary, since estrogenic compounds have the potential to increase the risk of endometrial cancer in women with intact uterus or the risk of breast cancer. Future animal studies are also warranted to better define the estrogenic efficacy of *Glyzyrrhiza* species *in vivo*.
